# Delayed Antarctic melt season reduces albedo feedback

**DOI:** 10.1093/nsr/nwad157

**Published:** 2023-05-27

**Authors:** Lei Liang, Huadong Guo, Shuang Liang, Xichen Li, John C Moore, Xinwu Li, Xiao Cheng, Wenjin Wu, Yan Liu, Annette Rinke, Gensuo Jia, Feifei Pan, Chen Gong

**Affiliations:** Key Laboratory of Digital Earth Science, Aerospace Information Research Institute, Chinese Academy of Sciences, Beijing 100094, China; International Research Center of Big Data for Sustainable Development Goals, Beijing 100094, China; Key Laboratory of Digital Earth Science, Aerospace Information Research Institute, Chinese Academy of Sciences, Beijing 100094, China; International Research Center of Big Data for Sustainable Development Goals, Beijing 100094, China; Key Laboratory of Digital Earth Science, Aerospace Information Research Institute, Chinese Academy of Sciences, Beijing 100094, China; International Research Center of Big Data for Sustainable Development Goals, Beijing 100094, China; Institute of Atmospheric Physics, Chinese Academy of Sciences, Beijing 100029, China; College of Global Change and Earth System Science, Beijing Normal University, Beijing 100875, China; Arctic Centre, University of Lapland, Rovaniemi 96101, Finland; CAS Center for Excellence in Tibetan Plateau Earth Sciences, Beijing 100101, China; Key Laboratory of Digital Earth Science, Aerospace Information Research Institute, Chinese Academy of Sciences, Beijing 100094, China; International Research Center of Big Data for Sustainable Development Goals, Beijing 100094, China; School of Geospatial Engineering and Science, Sun Yat-sen University, Guangzhou 519082, China; Key Laboratory of Digital Earth Science, Aerospace Information Research Institute, Chinese Academy of Sciences, Beijing 100094, China; International Research Center of Big Data for Sustainable Development Goals, Beijing 100094, China; College of Global Change and Earth System Science, Beijing Normal University, Beijing 100875, China; Alfred Wegener Institute Helmholtz Centre for Polar and Marine Research, Potsdam 14473, Germany; Institute of Atmospheric Physics, Chinese Academy of Sciences, Beijing 100029, China; Department of Geography, University of North Texas, Denton, TX 76203, USA; Key Laboratory of Digital Earth Science, Aerospace Information Research Institute, Chinese Academy of Sciences, Beijing 100094, China

**Keywords:** Antarctic ice sheet, snowmelt, season delay, sea ice, solar irradiance

## Abstract

Antarctica's response to climate change varies greatly both spatially and temporally. Surface melting impacts mass balance and also lowers surface albedo. We use a 43-year record (from 1978 to 2020) of Antarctic snow melt seasons from space-borne microwave radiometers with a machine-learning algorithm to show that both the onset and the end of the melt season are being delayed. Granger-causality analysis shows that melt end is delayed due to increased heat flux from the ocean to the atmosphere at minimum sea-ice extent from warming oceans. Melt onset is Granger-caused primarily by the turbulent heat flux from ocean to atmosphere that is in turn driven by sea-ice variability. Delayed snowmelt season leads to a net decrease in the absorption of solar irradiance, as a delayed summer means that higher albedo occurs after the period of maximum solar radiation, which changes Antarctica's radiation balance more than sea-ice cover.

## Teaser

The Antarctic snowmelt season shows a delay over the satellite record. Decreasing spring and early summer surface temperature leads to delay of snowmelt onset, while the melt season ends later mainly due to enhanced ocean-atmosphere heat exchange. The delay in snowmelt season results in reduction of net surface solar radiation.

## INTRODUCTION

Large rising and falling trends in regional surface air temperature (SAT) occur in Antarctica [1,2]. For example, over the second half of the twentieth century, West Antarctica and the Antarctic Peninsula warmed more than twice as fast as the global average [3–6] but SAT in East Antarctica decreased. Strong regional changes are typical of the polar regions where ice-albedo feedbacks can amplify warming [4,7,8], while stratospheric ozone depletion and extreme decadal variability can even induce regional cooling [9–12]. Fluctuations in Antarctic SAT may also occur because of the continent's sensitivity to tropical forcing [9,13–18], the strength and position of the circumpolar westerly winds [11,19–22] and its exposure to the relatively mild and moist oceanic air mass intrusions along its coastline [23–27].

The direct impact of these SAT changes on the Antarctic ice sheet is most important in the Antarctic summer when surface melting can occur. Here, we examine temperature changes in the summer seasons at the Antarctica ice shelves from satellite proxies and weather observations. Our aim is to examine the causal framework linking changing SAT and surface conditions, which involves atmospheric, oceanic and sea-ice variability.

## RECORDS OF TEMPERATURE CHANGES FROM THE ANTARCTICA ICE SHEET

**Figure 1.**
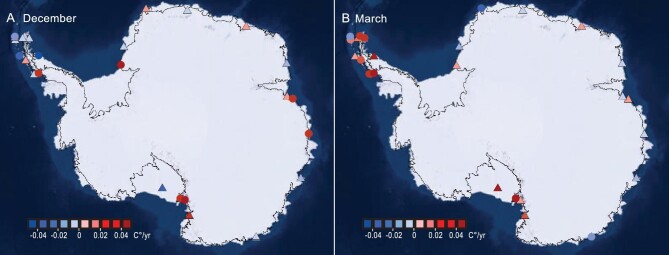
Trends in AWS 2-m air-temperature data (marked by triangles and circles) are shown for (A) December and (B) March. The colorbar denotes the temperature change per year over the period 1979–2020. The circular symbols represents statistically significant trends at the 95% level.

We use time series of *in situ* 2-m air-temperature data recorded by 32 long-duration automatic weather stations (AWSs) in Antarctica (Supplementary Table S1). Fifteen out of 32 of the AWS temperature data sets show a statistically significant decline for December (1979–2020; Fig. 1A) temperature, whereas in March, 15 out of the 32 AWSs show statistically significant rises (Fig. 1B). Therefore, there are indications of a delaying trend in the timing of the Antarctic summer over the last four decades.

## IMPACT OF TEMPERATURE CHANGE ON ICE-SHEET SNOWMELT SEASON

The start and end of the melt season in Antarctica vary depending on the region and can be influenced by factors such as latitude, altitude and local weather patterns. However, in general, the melt season in Antarctica usually begins in November and lasts until February. During this time, temperatures increase and the sunlight becomes more intense, causing some of the snow and ice to melt. The extent and thickness of sea ice decrease through the snowmelt season. Passive microwave remote sensing can be used to retrieve the snowmelt dynamics of the Antarctic ice sheet under all weather conditions [12]. The microwave brightness temperature (Tb) is very sensitive to changes in the physical characteristics of the ice-sheet surface, such as snowfall, snow age, snowmelt and snow density and densification [13–15]. However, the temporal scale at which the appearance of liquid water affects Tb is much shorter than the other factors. Therefore, the transition from dry snow to wet snow (liquid water, ice and air) yields a distinct signature: a sharp and abrupt increase in Tb, which is detectable by microwave sensors at frequencies of >10 GHz (see SI Discussion). Previous analyses using scanning multichannel microwave radiometer (SMMR), special sensor microwave/imager (SSM/I) and special sensor microwave imager/sounder (SSMIS) brightness temperature data have focused on variations in the snowmelt area and its duration across the ice sheet [4,16–18]. Although the melt extent (ME) and melt index (MI) [12] have been decreasing over Antarctica, both positive and negative trends exist in different regions [4,16–18]. Tedesco [19] suggests that the response of surface snow melt to climate forcing means that a 1°C increase in the average snowmelt season SAT corresponds to an average MI increase of ∼2 × 10^6^ km^2^ per day. In contrast to these earlier analyses, we use the Tb data (26 October 1978–30 June 2020) from SMMR, SSM/I and SSMIS processed using a machine-learning algorithm for ice-sheet snowmelt detection. We then characterize the trends, spatial patterns and interannual variability in the timing of the Antarctic ice-sheet-melt season—that is, the dates of melt onset and ending.

**Figure 2.**
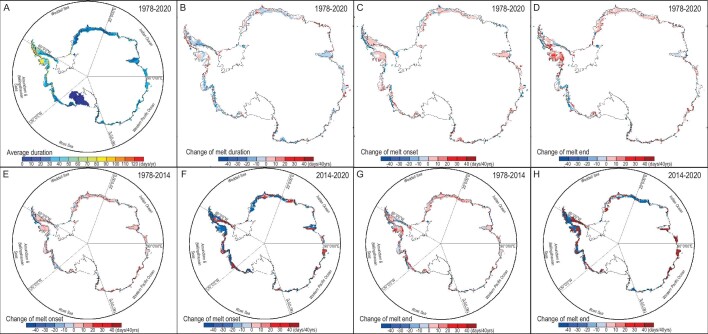
Temporal and spatial variation of snowmelt in Antarctica ice sheet derived from the SMMR and SSM/I data during 1978–2020. Reddish colors show areas with rates that are greater than zero and are significant at the 95% level, indicating that the onset or end date was delayed, whereas blueish colors indicate that the onset date or end date was significantly advanced. (A) Distribution of the average number of melt days per year on the Antarctic ice sheet during 1978–2020. Distribution of the regions where the trends in snowmelt (B) duration, (C) onset date and (D) end date are significant at the 95% level. 54% of the snowmelt area was shortened in duration by ≥0.1 days per year, while 29% of the snowmelt area extended their duration by >0.1 days per year. 62% (of which 67% have a 95% significant trend) and 52% (of which 57% have a 95% significant trend) of the snowmelt area experienced delays in onset and termination dates by >0.1 days per year, respectively. Distribution of the regions where the trends in snowmelt onset date during (E) 1978–2014 and (F) 2014–20 and end date during (G) 1978–2014 and (H) 2014–20 are significant at the 95% level. The Ross Ice Shelf region was not included in the statistical analysis because of the infrequent snowmelt in these areas.

Figure 2 presents the characteristics of the Antarctic melt season and its changes over 1978–2020. Melt duration has shortened by ∼63% and extended by ∼37% of the total melting region (Fig. 2B), with ∼54% of the snowmelt areas shortened in duration by more than −0.1 days per year and 29% of the snowmelt areas lengthened their duration by >0.1 days per year. However, the small differences in melt duration obscure the fact that 67% of the snowmelt areas experience delays in onset and 65% delays in the termination dates. Moreover, most of the snowmelt areas in Antarctica have experienced a delay in both the melt onset (Fig. 2C) and end dates (Fig. 2D). These results indicate that the accumulated delays in both the melt onset and end dates over the 40-year observational period amount to 10%–15% of the whole summer melt period and that the Antarctic summer is not only ‘coming late’ but also ‘ending late’.

The delays in melt onset and end dates are consistent with the temperature decreases in December and increases in March as recorded by the 32 AWSs (Fig. 1). We explore the relationship between temperature and snowmelt date (Supplementary Fig. S1A–F) through maximum covariance analysis (MCA) [28]. Using MERRA and ERA5 2-m Temperature data sets, the first mode of 2-m temperature with snowmelt onset captures >75% of the squared covariance. In contrast, the first mode of 2-m temperature with snowmelt end captures <50% of the squared covariance (Supplementary Fig. S1A–D). However, the first mode of ERA5 sea surface temperatures (SST) with snowmelt onset captures <50% of the squared covariance, but the first mode of SST captures 55% of the squared covariance with snowmelt end (Supplementary Fig. S1E and F). These analyses point towards the delay of the onset of freeze–thaw being driven by the atmosphere, whereas the delay of the end of freeze–thaw may be driven by the ocean. Therefore, we next analyse the atmosphere, sea ice, ocean and the melt date.

## MECHANISMS DRIVING THE SNOWMELT SEASON DELAY

Antarctic snowmelt areas are generally in the climatically complex coastal regions. Mechanisms driving the snowmelt season and surface temperature changes may involve changes in atmospheric or oceanic circulations, changes in sea-ice extent and changes in turbulent and radiative fluxes.

**Figure 3.**
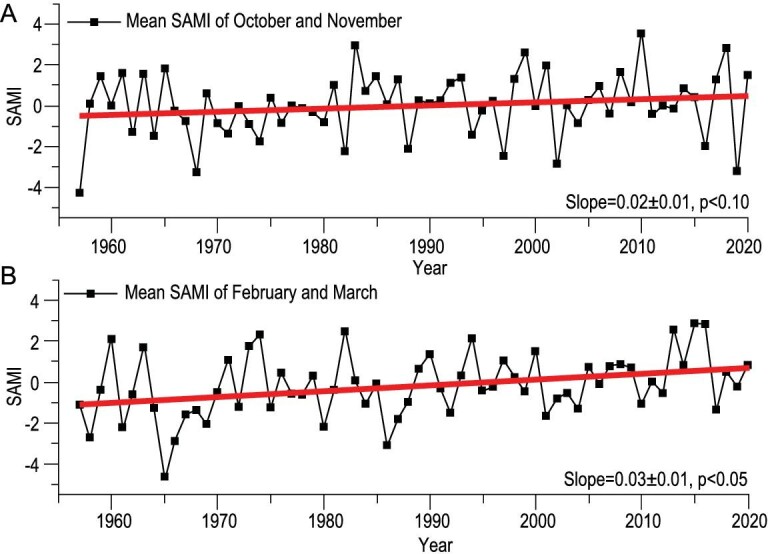
Interannual variation of SAM index (SAMI) (1957.1–2020.12 obtained from https://legacy.bas.ac.uk/met/gjma/sam.html). (A) Melt onset (October and November) and (B) end date (February and March) with trend lines fitted for 1957–2020 (red line). The estimated slopes and its 95% confidence intervals are shown.

A potential driver is the Southern Annular Mode (SAM) [20,21,29], which represents the gradient of the sea-level pressure between mid- and high latitudes of the southern hemisphere, and hence poleward or equatorward shifts in the westerly jet. A positive phase of the SAM indicates abnormally high sea surface pressures in the mid-latitudes that drives the westerly jet poleward, usually causing a decline in surface temperatures near the pole. The SAM exhibits a positive trend, significant at the 90% level, for the beginning of the summer melt season (Fig. 3A) and a 95% significant positive trend level for the melt end date (Fig. 3B). Modeling studies suggest that the trend of the SAM is likely due to stratospheric ozone depletion [22] and natural variability [30]. Furthermore, northern hemisphere and tropical SST variability may have played a triggering role in the shift of the SAM into its positive phase [31,32] and the adjustment of the southern hemispheric atmospheric circulation, or it may simply be the internal variability of the system [33]. This trend towards a positive phase of the SAM may potentially contribute to the cooling in East Antarctica but relative warming over the more northerly regions of the Antarctic Peninsula [22,23].

Another candidate that may drive the change in the Antarctic snowmelt season is the sea-ice cover. The thermal capacity of the ocean is an important heat source of the atmosphere over Antarctica. Sea ice affects the radiation balance through the sea ice-albedo feedback. Moreover, sea ice blocks the exchange of heat between the ocean and the atmosphere, lowering the vertical heat transfer by two orders of magnitude [26]. Antarctic sea-ice changes have been attributed to the adjustment of both the SAM [24] and the Amundsen Sea Low, which may be further driven by the teleconnections triggered by tropical interannual and decadal variabilities [28,34–37]. In addition, a large fraction of sea-ice variance can be explained by Rossby wave-like structures in the Drake Passage region [25].

**Figure 4.**
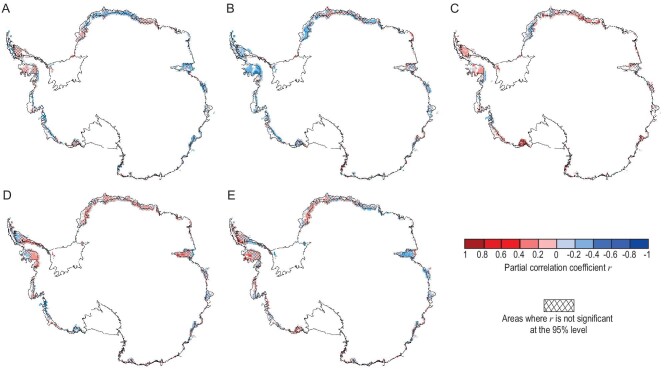
Correlation between melt timing and SAM, SIC and turbulent heat flux. Spatial distribution of partial correlation coefficients between melt end date and (A) SAM, (B) SIC and (C) turbulent heat flux. Spatial distribution of partial correlation coefficients between melt onset date and (D) SAM and (E) SIC. The continent and oceans around Antarctica are divided into five regions (see Fig. 1A), then, for each region, we calculate the partial correlation coefficients between melt dates over continent and regional-averaged SIC, regional-averaged turbulent fluxes over sea. Hatching shows individual grid cells where the trend was not significant at the 95% level. The correlation analysis is performed on detrended data.

The increasing trend in Antarctic sea ice over the satellite record is the integration of large but regionally contrasting trends and other regions that show very little net change. In particular, the sea ice extent (SIE) over the Amundsen and Bellingshausen Seas exhibits a strong decreasing trend (Supplementary Fig. S2). This regional feature of the SIE melting trends also contributes to the regionality of the Antarctic snowmelt onset and end dates. Antarctic sea ice increased from the late 1970s until 2014 (before its abrupt retreat after 2015) for all months during the Antarctic snowmelt season (from October to the March of the following year; Supplementary Fig. S2A–C) [5,6,38] particularly in October (second-greatest increase) and December (fourth-greatest). As a result, the melt season onset was delayed around the entire Antarctic coast from 1979 to 2014 (Fig. 2E). The snowmelt onset delay over this period is consistent with the reduction in heat exchange from ocean to atmosphere expected by extended sea-ice cover. In contrast, a rapid reduction in Antarctic sea ice [39] after 2015 led to earlier snowmelt onset (Fig. 2F), again consistently with increased heat flux from the ocean to the atmosphere caused by the sea-ice decline in the period. While the integrated change trend over Antarctica shows earlier snowmelt onset and delayed snowmelt end after 2015, comparing Fig. 2E and G, there are trend reversals in the change in melt onset and melt end for some regions and compensating increases in trends in other regions. Sea ice plays an important role in regulating the heat exchange between the ocean and the atmosphere, and can have a significant impact on the Antarctic snowmelt freeze–thaw date changes. Sea ice acts as an insulator, preventing heat transfer between the ocean and the atmosphere. As sea-ice extent decreases, more heat is transferred from the ocean to the atmosphere, warming the air and increasing the likelihood of melt events. The changes in Antarctic sea ice since 2015 have been complex. While overall trends indicate a decrease in sea-ice extent, there have been some regional variations and short-term fluctuations. Changes in sea ice after 2015 led to shifts in ice-sheet-melt onset and end timing. Figure 4B and E shows the spatial distribution of partial correlation coefficients between melt date and sea-ice concentration. In the Amundsen Sea and western Peninsula, melt onset has a significant negative correlation with sea-ice concentration, while melt end has a significant positive correlation with sea-ice concentration. The reduction in sea-ice concentration in this region after 2015 led to an earlier onset and a later melt end.

**Figure 5.**
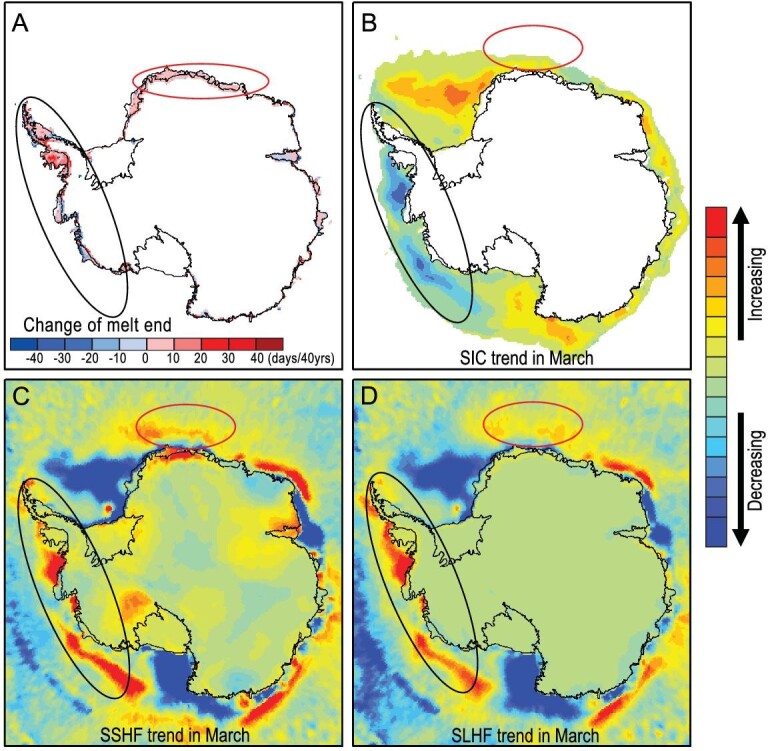
Relationships between March trends in four factors. March trends in (A) end of melt season, (B) sea-ice concentration (SIC; 1978.10–2020.12, obtained from https://nsidc.org/data/nsidc-0051/versions/2), (C) sensible heat flux at the surface (SSHF) and (D) latent heat flux at the surface (SLHF). Surface sensible and latent heat fluxes are from ERA5 reanalysis data, https://cds.climate.copernicus.eu/cdsapp#!/dataset/reanalysis-era5-land?tab=form. The two vertical fluxes are positive upwards. In the Bellingshausen and Amundsen Seas and western Peninsula (black oval), the sea-ice concentration decreased markedly in this season during 1978–2020; the SLHF and SSHF release also shows an increase, meaning increased heat exchange between the ocean and the atmosphere. Red ovals mark Queen Maud Land where similar patterns occur.

At the end of the melt season, the sea-ice extent around Antarctica is at its minimum and ocean–atmosphere heat exchange is at its greatest, meaning that ocean conditions have their greatest impact on the atmosphere. This behavior is clearly visible in the partial correlation between melt season end dates and turbulent heat flux, and in the anti-correlation with sea-ice concentration (SIC) around the entire coast (Fig. 4B and C). Although Antarctic sea ice has generally increased, Supplementary Fig. S2D and E shows that the SIC in Bellingshausen and Amundsen Seas decreased during February and March, i.e. especially at the end of the melt season (Supplementary Fig. S2D and E and black circle in Fig. 5B). The sea-ice decline led to increased heat released from the ocean to the atmosphere during March via turbulent heat fluxes, shown as sensible heat (Fig. 5C) and latent heat (Fig. 5D). A similar feature can also be seen off the coast of Queen Maud Land. Both these regions show delayed ending of the melt season (Fig. 5A). However, regions where turbulent heat fluxes are not increasing, such as off Victoria Land, show no trend towards delayed melt season termination.

To test the nature of the relationships between the potential causal factors, i.e. SAM, SIC and turbulent heat flux, and the melt season timing, we performed Granger-causality tests. A time series is said to Granger-cause a target time series if it better explains future values of the target than does the target's own past values. Since this test utilizes the time-ordering of the data, it is a more useful in detecting causality than correlation analysis.

**Figure 6.**
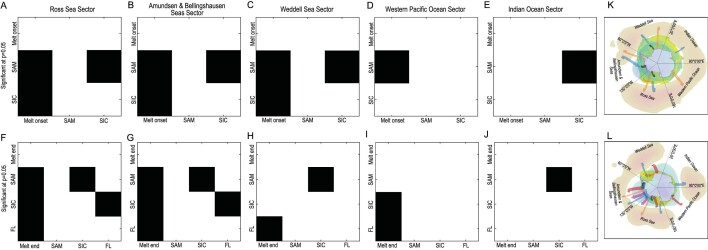
Grange-causalities test between melt timing and SAM, SIC and turbulent heat flux in the five sectors: panels A–E show the causalities of melt onset date, SAM and SIC in the five sectors, which indicate that the time series of SAM and SIC are useful in forecasting melt onset date; panels F–J show the causalities of melt end date, SAM, SIC and turbulent heat flux in the five sectors, which also indicate that the time series of SAM, SIC and turbulent heat flux (FL) are useful in forecasting the melt end date. In other words, SAM, SIC and turbulent heat flux cause (or, strictly speaking, Granger cause) the delay in the melt season. Note: the continent and ocean around Antarctica are divided into five regions (see Fig. 1A), then, for each region, Granger causalities were tested between melt dates over the land and SIC, turbulent fluxes over the sea. Panels (K) and (L) visualize the causalities: orange arrows show the causalities between the SAM and melt date; blue arrows between the SAM and SIC; black arrows between the SIC and melt date; red arrows between the turbulent heat flux and melt date; and purple arrows between the SIC and turbulent heat flux.

Although our postulated mechanisms as described above work on subannual timescales, which we cannot directly examine with only one melt season available each year, we can study the multi-annual persistence of Granger causality by using the full 43-year-long records. Figure 6A–E shows the outcomes of the Granger-causalities test between the factors in five geographic sectors. In the Ross Sector and Amundsen and Bellingshausen Seas Sector, the SAM Granger-causes the SIC (i.e. that is changes in the SAM provide statistically significant (*P* < 0.05) forecasting for SIC); the SIC Granger-causes turbulent heat flux; all three variables Granger-cause a delay in the melt season end time. This is consistent with the arguments and mechanisms proposed earlier, but the causality relationships are significant over multi-annual and multi-decadal timescales in addition to simple seasonal relations.

In contrast, during the onset of summer, the sea-ice extent is near its maximum in November and the ocean–atmosphere heat exchange is greatly reduced [26] (Supplementary Fig. S2A–C). Therefore, other factors, such as atmospheric circulation, can play larger roles in determining air temperatures. The SAM monthly index shows a positive trend for all months from October to March (Fig. 3A and B). The melt onset date is correlated with the SAM and the SIC in the majority of melt areas (SAM: 61% of which 41% have a trend significant at the 95% level and SIC: 55% of which 38% are significant at the 95% level; Fig. 4D and E), indicating that as the SAM and the SIC increase, the melt onset date is delayed. Regionally, the Amundsen, Bellingshausen Seas and Queen Maud Land show the highest correlations and are regions where the change in onset date is the largest (Fig. 2C) while areas with weak or negative correlation tend to show smaller changes in melt onset date. The trend of the SAM towards a positive phase leads to increases in eastward wind stress over the Southern Oceans and a shift in the band of maximum wind stress southwards, which has had a major impact on ocean temperatures [40], leading to a cooling of the atmosphere. This cooling dominates conditions at the start of the snow melt season when oceanic heat exchange is minimal. We again demonstrate that the time series supports the causal rather than simple correlations between the variables using the Granger-causality tests shown in Fig. 6. In the Ross Sector, Amundsen and Bellingshausen Seas Sector and Weddell Sea Sectors, the SAM is found to significantly Granger-cause the SIC; and both the SAM and the SIC Granger-cause changes in melt onset. Thus, the increased SIC with the positive phase of the SAM can Granger-cause the delay in the melt onset date.

## POTENTIAL IMPACTS OF THE SNOWMELT SEASON DELAY

The timing of surface melt affects Antarctic radiation balance and climate via the change in albedo. To investigate the impact of the delay of the snowmelt season on the change in solar radiation, we estimated the annual changing rates of surface net solar radiation using the rates of the snowmelt onset and end dates, snowmelt area, insolation and snow albedo. Albedos of dry snow are 0.9–0.98 while for wet snow they are 0.6–0.8 [41,42]. The observed delay in the snowmelt season decreases the annual surface net solar radiation of Antarctica by −5 ± 3 × 10^18^ J per year (averaging to −0.26 ± 0.14 W/m^2^ per year in the snow melt area) or −0.3% per year given an annual average surface net solar radiation of 1.54 × 10^21^ J (calculated by using ERA5 reanalysis data [43]). We ignore the impact of changes in blue ice areas on solar radiation as blue ice occupies <4% of the snowmelt area [44]. The reduction in surface net solar radiation on the snowmelt area caused by the delay of the snowmelt season is larger than that caused by the observed increases in sea ice, which we estimate as −0.19 ± 0.31 W/m^2^ per year or −0.23% per year (the range of sea-ice albedo is 0.6–0.8 and the albedo of sea water is 0.06 [42,45]). Thus, the delay in the snowmelt season effectively causes more than twice the total change in radiation balance as by the widely discussed and speculated on changes in Antarctic sea ice.

Prognostic simulations of future Antarctic climate suggest a rise in precipitation as the air warms. Increased snowfall would raise the snow albedo, reducing surface temperatures and surface melt [18]. Recent work [7,10,11] on the surface melt and hydrology of Antarctica has not considered the delayed melt season phenomenon that we report, which depends on the complex interaction of ocean and atmospheric drivers. In contrast with previous studies [46], our new snow melt detection method (see ‘Methods’ and SI Discussion) finds significant trends in the timing of the melt season, as well as its spatial variability. These trends suggest that the observed surface hydrology may well change in response to variations in melt sources. Since surface melt plays a crucial role in hydrofracturing ice shelves, the melt season delay is likely to be an important factor in future Antarctic mass balance and ice-sheet stability [9,47,48]. Better incorporation of the snowmelt processes within climate and ice-sheet models will be useful for improving projections of regional climatic change, ice-sheet mass balance and sea-level rise, and the satellite era observations of melt season change will provide a valuable validation data set for such models.

## METHODS

The research framework of this study contains four main aspects as outlined in Supplementary Fig. S3.

### Snowmelt detection method based on machine learning

Using 18- and 19-GHz horizontal polarization channel data of the SMMR, SSM/I and SSMIS, respectively, we followed these procedures. (i) The time-series brightness temperature values of each pixel are used to differentiate the melt area and non-melt area. Traditional methods [4,15–17,19] usually extract abrupt signals around the melt season, with a threshold value to identify melting events. However, their accuracy is limited because snow in different locations can have distinct characteristics and therefore the melting signals may vary significantly spatially. We improved the accuracy of the detection using a novel method based on modern machine-learning techniques [49]. The architecture of the model is shown in Supplementary Fig. S4A and B. We employed a neural network with three convolution blocks combined with fully connected layers to capture meaningful fluctuations in the daily Tb series. Our method makes use of both local and global fluctuation patterns during the entire year since melt happens gradually along with changes in the grain size, density and crystal structure of the surface snow and ice, all of which can change the emissivity. (ii) In the preprocessing step, we normalized the Tb series for easier training of the network. The air-temperature data from AWS was taken as ‘ground truth’. Nine hundred and fifty-six samples were selected with labels derived from the corresponding AWS data, 80% of which were used for training and 20% for validation. Prior to training, we performed a quality control of AWS data [50]. Some spurious observations may appear in the AWS data set, e.g. the temperature sensor may heat up because of direct and/or diffuse solar radiation in spite of the radiation shield [50], creating errors of up to several degrees. Therefore, we rejected temperature data outside three standard deviations from the mean during the selected time periods. After training for 60 epochs, the new method achieved 93% overall validation accuracy (see Supplementary Fig. S4C) which is >10 percentage points higher than the widely adopted method based on the generalized Gaussian distribution [51]. This significantly benefits the timing accuracies for the onset and termination of the snow melt season. (iii) The model was then used to classify all the experimental data and locate the melting areas. (iv) The time-series brightness temperature values of each wet pixel were decomposed into multiscale components through a wavelet transform. (v) The local extrema of the wavelet transform modulus (modulus extrema), which indicate the strength of the edges caused by sharp transitions and the time when the sharp variations occur, were tracked and analysed across scales. (vi) For each pixel, a critical value was set as a melt signal to differentiate the sharp transition induced by the melting and refreezing processes from the other transitions caused by non-melt processes by variance analysis. (vii) Based on the principle of spatial autocorrelation, a spatial neighborhood operator was used to detect and correct possible errors brought about by noise pixels.

This method can determine not only whether an area experienced melt, but also when an area experienced melt by detecting and tracking strong and significant edges in the brightness temperature time-series curve. Because the snowmelt detection in each year is only based on Tb time series from that year, the detection results between the years are independent.

### Snowmelt method validation

We validate our method by comparing with the melt results obtained by the 27th Chinese national Antarctic expeditions (Supplementary Fig. S9), AWS station observations (Supplementary Table S2), remote sensing based on RADARSAT SAR and AMSR-E data, and also using the cross-polarized gradient ratio method [52] in Supplementary Table S3. The comparisons show that our new method with the data sets used here outperform other approaches (see SI Discussion).

### Granger causality

Assuming multiple jointly distributed vector-valued stochastic processes (‘variables’) ${x^i} = x_1^i,x_2^i, \ldots ,i = 1,2, \ldots $. We say that ${x^i},i = 2,3, \ldots $ does not Granger-cause *x*^1^ if and only if *x*^1^, conditional on its own past, is independent of the past of ${x^i},i = 2,3, \ldots $; intuitively, past values of *x^i^*, $i = 2,3, \ldots $ yield no information about the current value of *x*^1^ beyond information already contained in the past of *x*^1^ itself. If, conversely, the past of *x^i^*, $i = 2,3, \ldots $ does convey information about the future of *x*^1^ above and beyond all information contained in the past of *x*^1^, then we say that *x^i^*, $i = 2,3, \ldots $ Granger-causes *x*^1^ [53]. In our Granger-causality test, the variable *x*^1^ is the melt end date or the melt onset date; the variables *x^i^, i* = 1, 2, 3 are the SAM, SIC and turbulent heat flux, respectively. We use their monthly values in October and November for melt onset, and in February and March for melt end. All the tested time series must be of the same length. Because we expect that regions closest to the coast have the greatest influence on melt conditions, we limit the region using a tentative threshold of 175 km from the coast line for the SIC and turbulent heat flux data. However, this means that the Antarctica Peninsula and the Bellingshausen and Amundsen Sea sectors have fewer data cells for SIC and heat flux than the number of melt season observations on land. To ensure that the same number of samples were used for the Granger test for all the variables, we randomly chose 2000 sample points (80% of the total) for the melt, SIC and heat flux data.

### New proposed parameters

To characterize the interannual variability in the timing of the Antarctic ice-sheet melt, three parameters that can detect the tendency of the snowmelt to changes are proposed based on the time-series regression analysis of the melt onset date, end date and duration. These parameters are the change rates of the melt onset date, end date and duration. In the analysis of melt onset and end date, the melt onset is the initial detected time of the presence of liquid water in the upper snowpack, which corresponds to the first significant upward edge on the daily Tb curve. The melt end is the last detected time of the presence of liquid water in the upper snowpack, which corresponds to the last significant downward edge on the daily Tb curve. The time that elapses between the first significant upward edge and the last significant downward edge defines the melt season. Moreover, 1 July and 30 June of the following year are defined as the first and last day, respectively, of the Antarctic year. The estimation process of the change rates in the snowmelt melt date is as follows (taking the change rates of the melt onset date as an example). (i) The annual snowmelt onset date for the entire Antarctic ice sheet for each of Antarctic years is derived using an ice-sheet freeze–thaw detection method based on machine learning. (2) A least-squares fitting is conducted and the linear trend of the annual melt onset date for each pixel is obtained (Supplementary Fig. S11). (iii) A change analysis based on linear trends is performed. A positive rate of change (positive slope; see Supplementary Fig. S11) in the snowmelt onset date indicates that the melt onset date has been delayed and a negative rate of change means that the melt onset date has advanced. Moreover, two other indicators are used to study the interannual temporal and spatial variability of the Antarctic ice-sheet snowmelt, namely the ME (km^2^) and the MI (day × km^2^) [12].

## Supplementary Material

nwad157_Supplemental_File

## Data Availability

The longest record of the microwave brightness temperatures from 26 October 1978 to 30 June 2020 spans 43 years and is derived from SMMR, SSM/I and SSMIS, downloaded from the National Snow and Ice Data Center (NSIDC) [54,55] and quality controlled by using several procedures: (i) corrections for long-term calibration drifts that result from changes in the operational behavior of the sensor and its electrical components; (ii) corrections for calibration drifts with respect to the sun-spacecraft (ecliptic) angle caused by variations in solar heating of the instruments at different positions in the orbit; (iii) statistical analysis on the brightness temperature data to look for possible calibration errors; (iv) along-scan adjustment using the Wentz radiative transfer model [56]. SSM/I data are missing for 28 days in December 1987; the snowmelt detection is thus incomplete and the 1987–88 season is not included in the analysis. For comparability among different platforms and sensors, the SMMR, SSM/I F11, SSM/I F13 and SSMIS F17 data were converted into the corresponding equivalent SSM/I F8 data using the linear regression method [46,57].
