# Supplementary material for: Delayed Antarctic melt season reduces albedo feedback
Source: Natl Sci Rev. 2023 May 27;10(9):nwad157. doi: 10.1093/nsr/nwad157 (PMC10411670; doi:10.1093/nsr/nwad157)
Supplement: nwad157_Supplemental_File [file nwad157_supplemental_file.doc]

**RESEARCH ARTICLE**

**EARTH SCIENCES**

**Delayed Antarctic melt season reduces albedo feedback**

Lei Liang^1,2^, Huadong Guo^1,2^, Shuang Liang^1,2^, Xichen Li^3^, John C. Moore^4, 5, 6, *^, Xinwu Li^1,2, *^, Xiao Cheng^7^, Wenjin Wu^1,2^, Yan Liu^4^, Annette Rinke^8^, Gensuo Jia^3^, Feifei Pan^9^ and Chen Gong^1^

^1^ Key Laboratory of Digital Earth Science, Aerospace Information Research Institute, Chinese Academy of

Sciences, Beijing 100094, China

^2^ International Research Center of Big Data for Sustainable Development Goals, Beijing 100094, China

^3^Institute of Atmospheric Physics, Chinese Academy of Sciences, Beijing100029, China

^4^College of Global Change and Earth System Science, Beijing Normal University, Beijing 100875, China

^5^Arctic Centre, University of Lapland, Rovaniemi 96101, Finland

^6^CAS Center for Excellence in Tibetan Plateau Earth Sciences, Beijing 100101, China

^7^School of Geospatial Engineering and Science, Sun Yat-Sen University, Guangdong 519082, China

^8^Alfred Wegener Institute Helmholtz Centre for Polar and Marine Research, Potsdam 14473, Germany

^9^Department of Geography, University of North Texas, Denton, TX 76203, USA

***Corresponding author** E-mail: john.moore.bnu@gmail.com, lixw@aircas.ac.cn


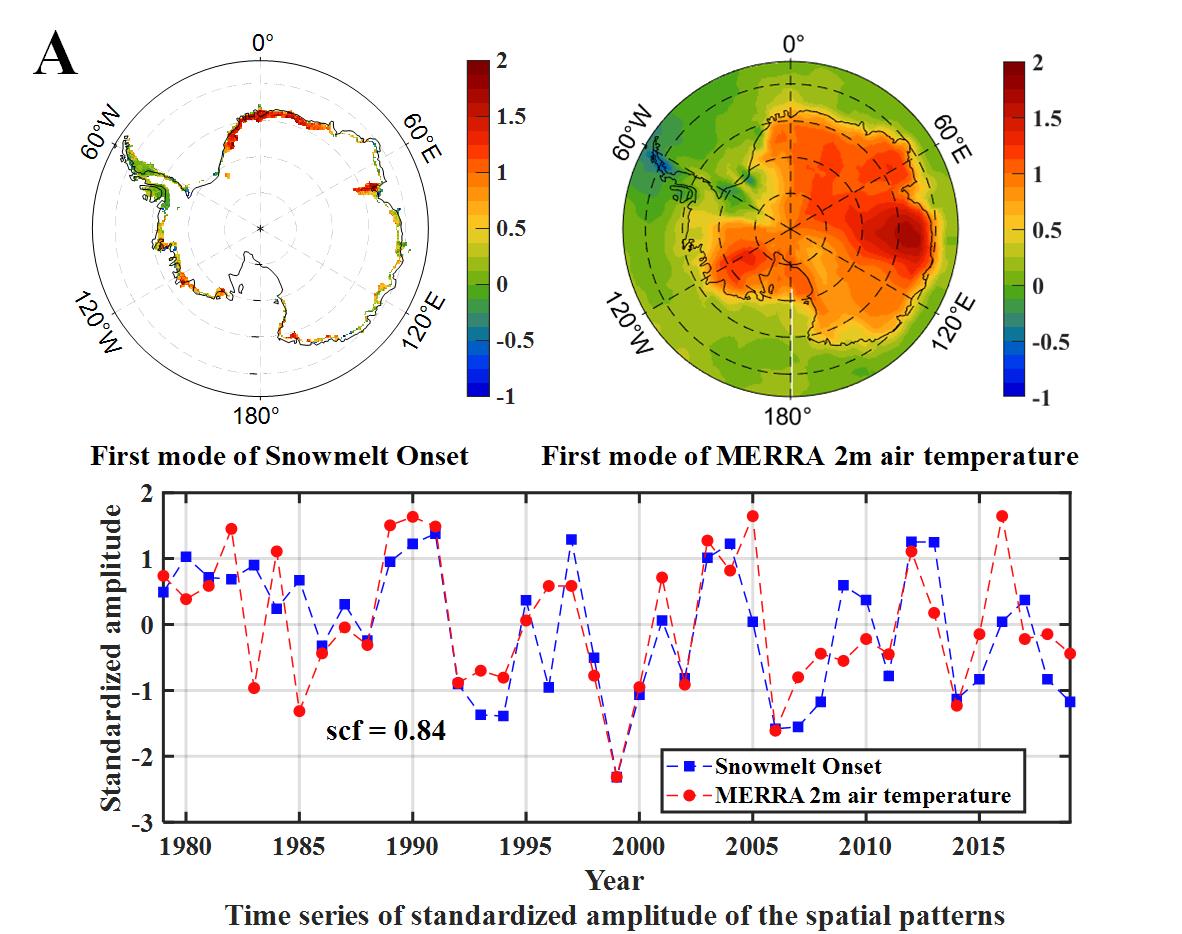

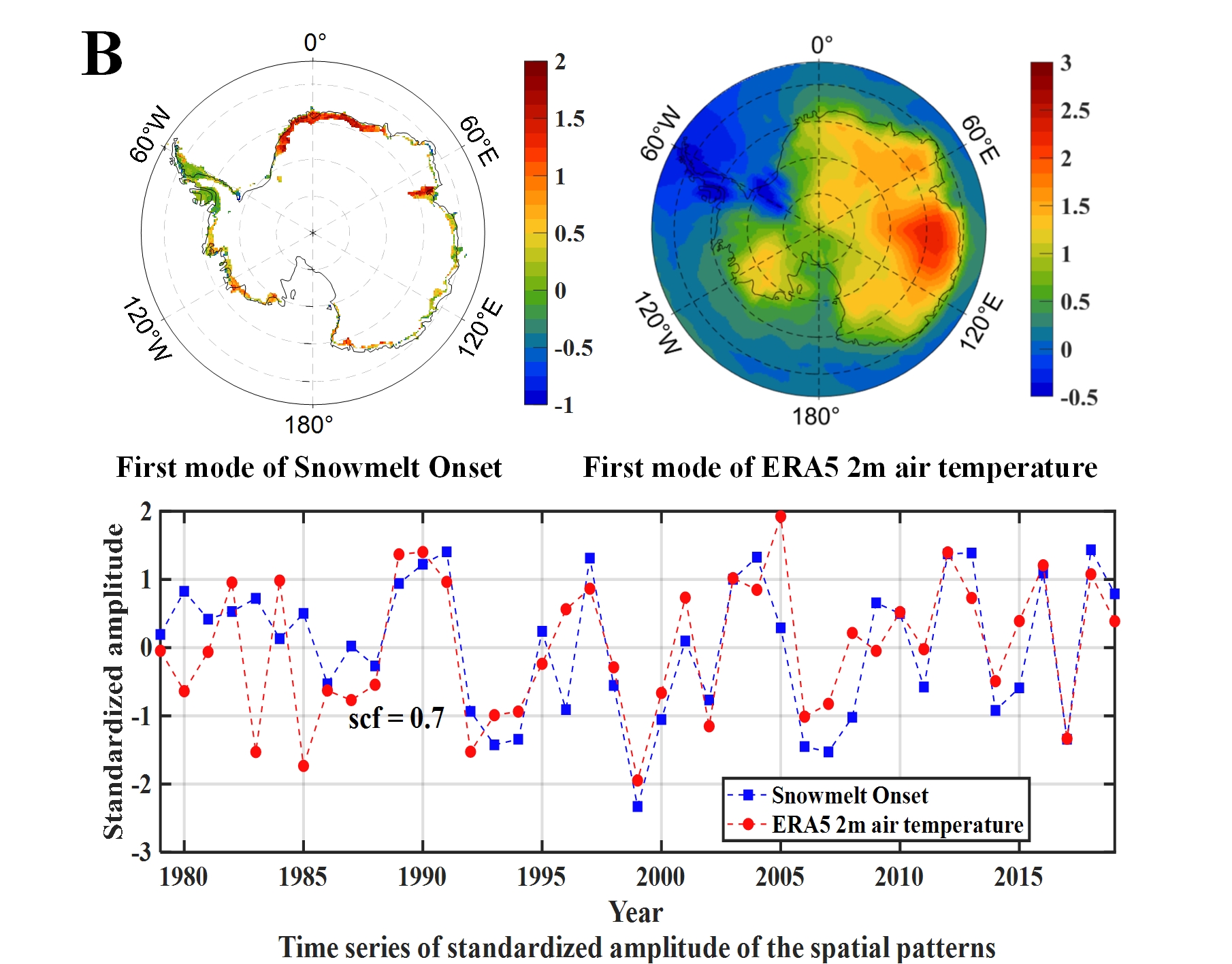


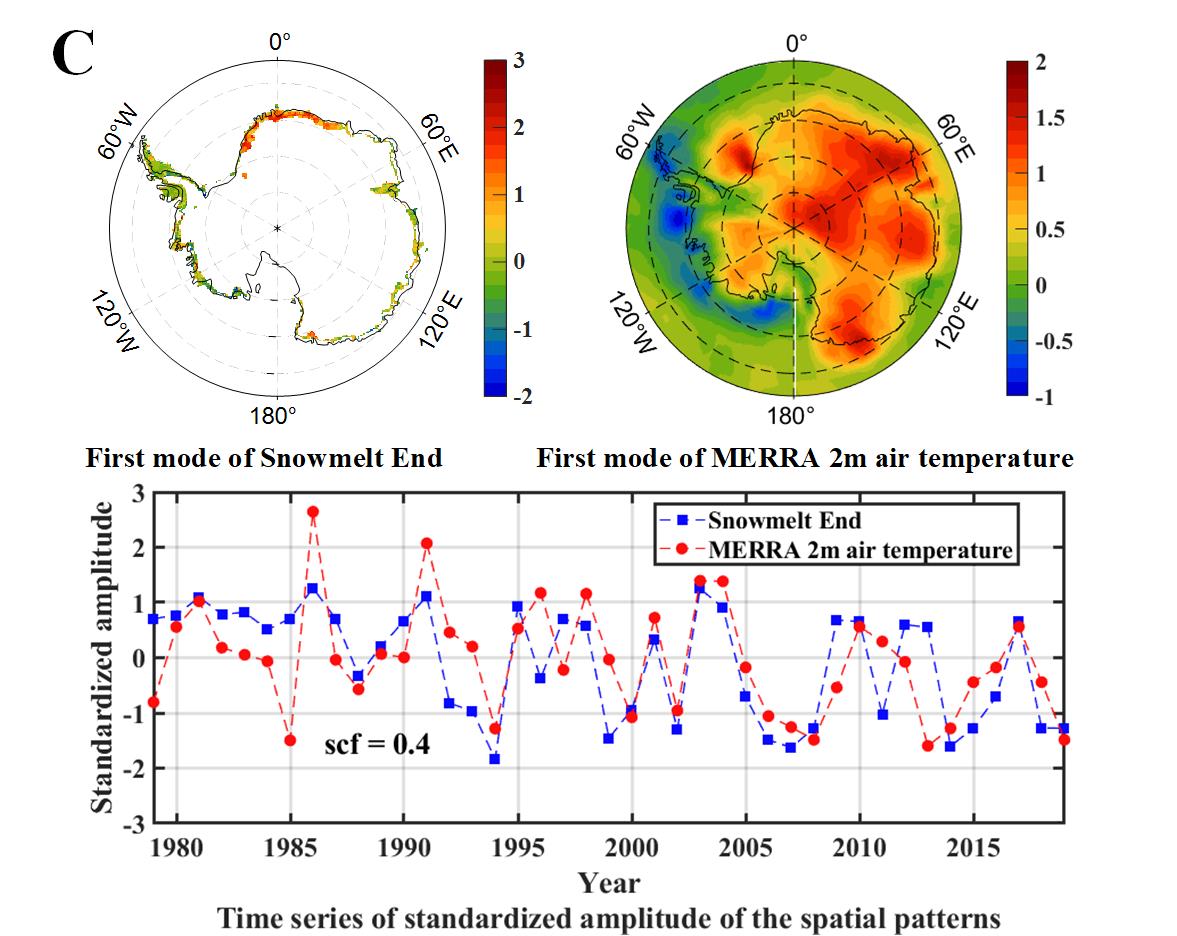

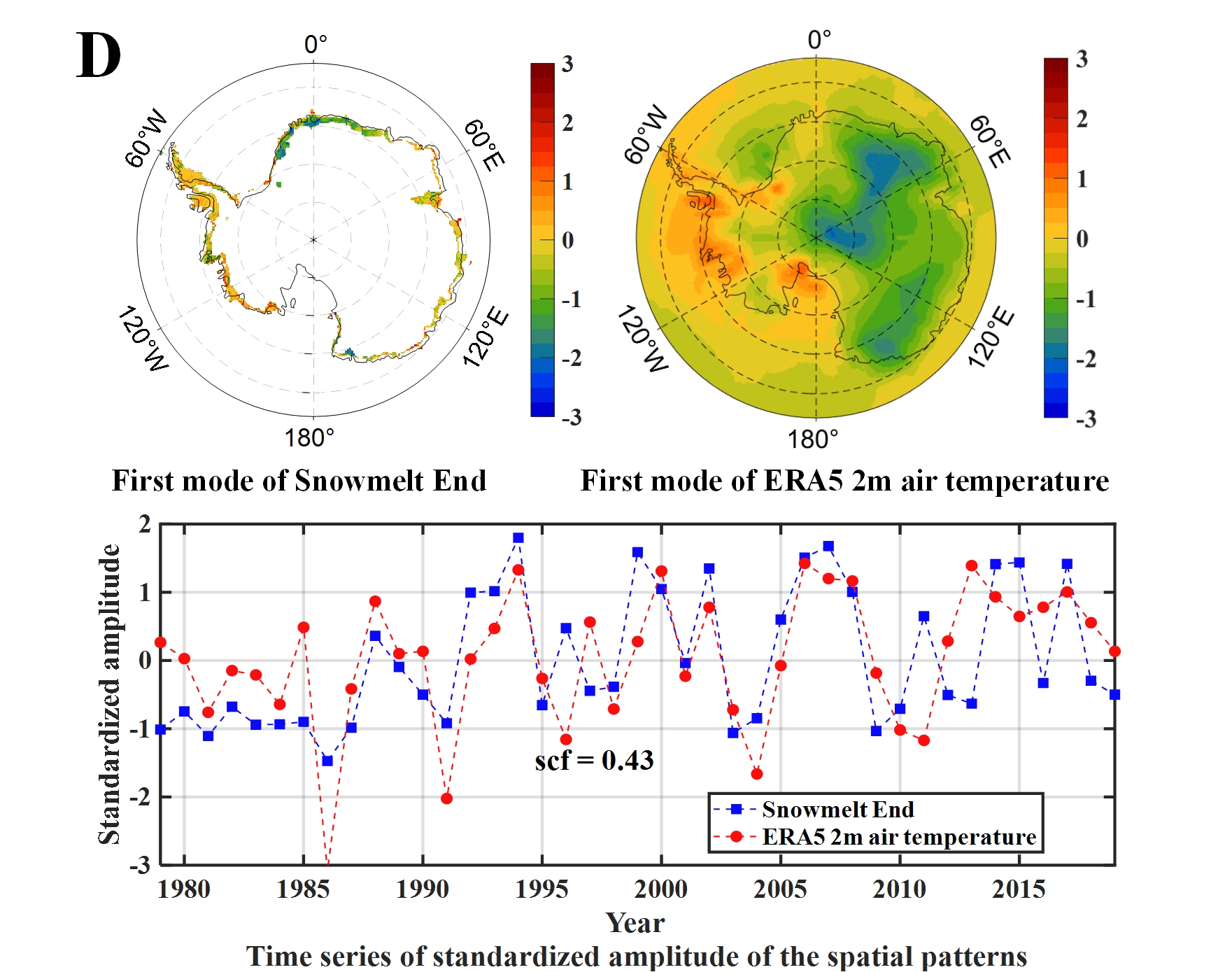


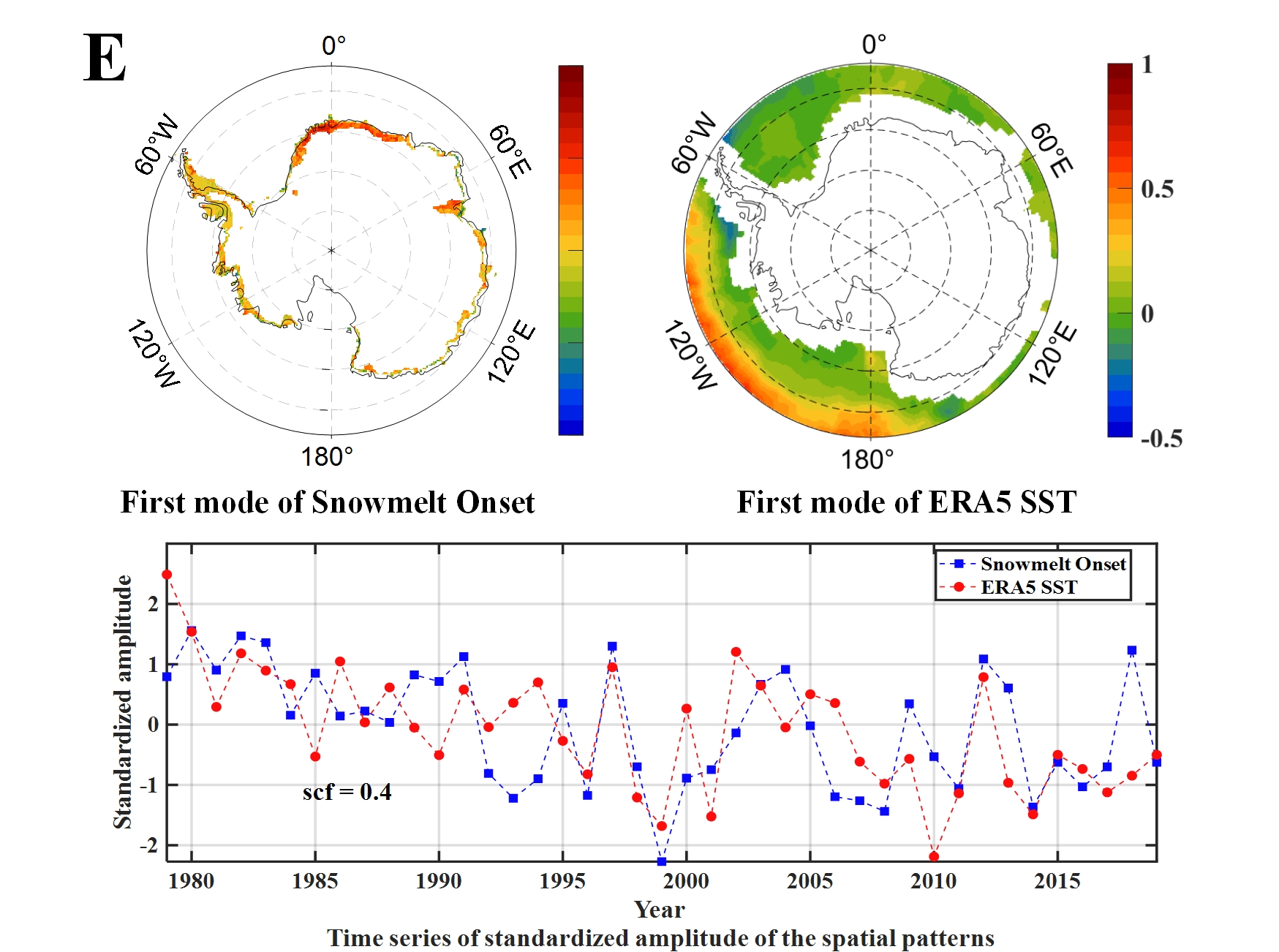

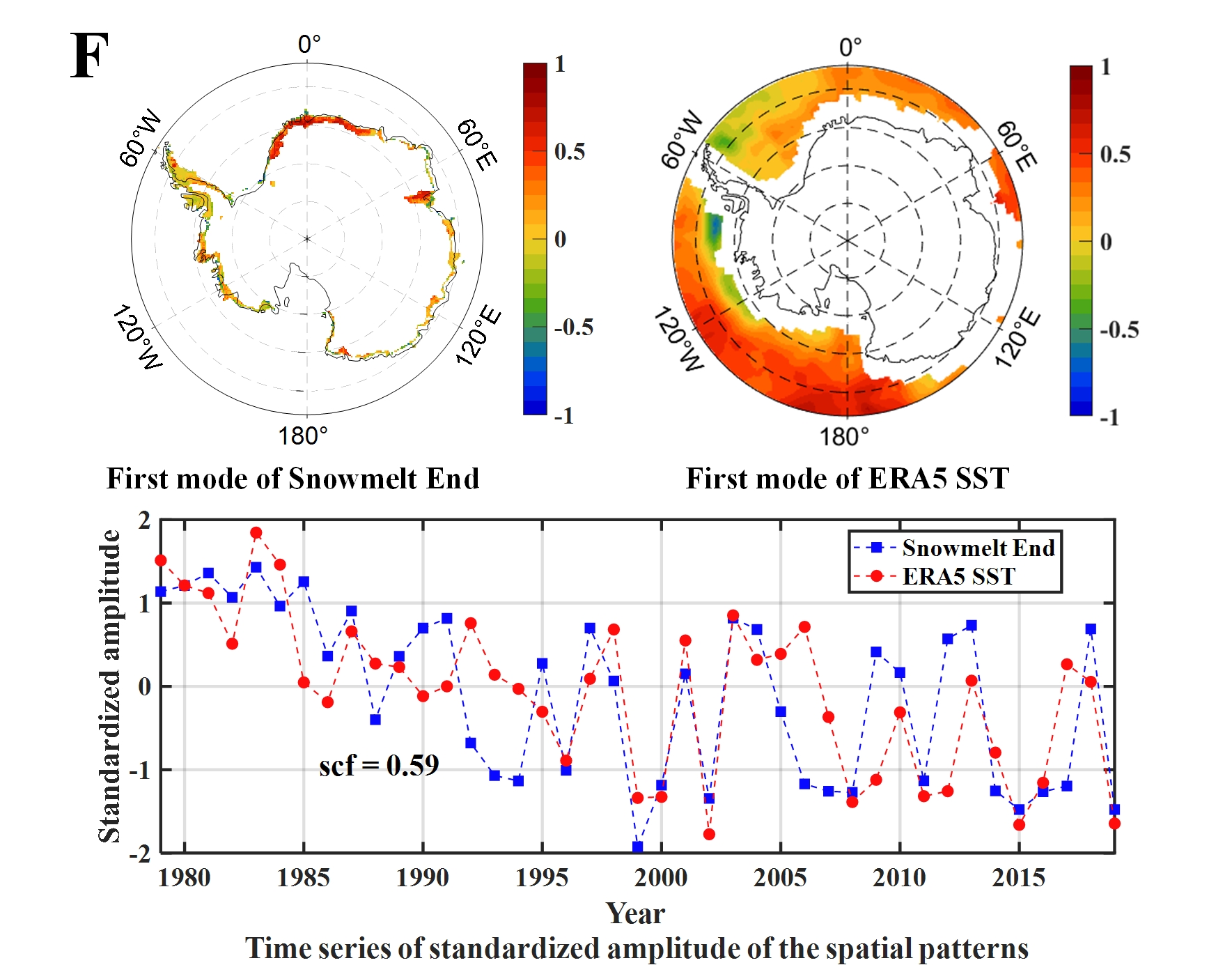


**Figure S1**. (A)The first mode of MCA, including the spatial patterns of MERRA 2 m temperature and melt onset, as well as their time series (unit free); (B) The first mode of MCA, including the spatial patterns of ERA5 2 m temperature and melt onset date, as well as their time series (unit free); (C) The first mode of MCA, including the spatial patterns of MERRA 2 m temperature and melt end date, as well as their time series (unit free); (D) The first mode of MCA, including the spatial patterns of ERA5 2 m temperature and melt end date, as well as their time series (unit free); (E) The first mode of MCA, including the spatial patterns of ERA5 SST and melt onset date, as well as their time series (unit free); (F) The first mode of MCA, including the spatial patterns of ERA5 SST and melt end date, as well as their time series (unit free).


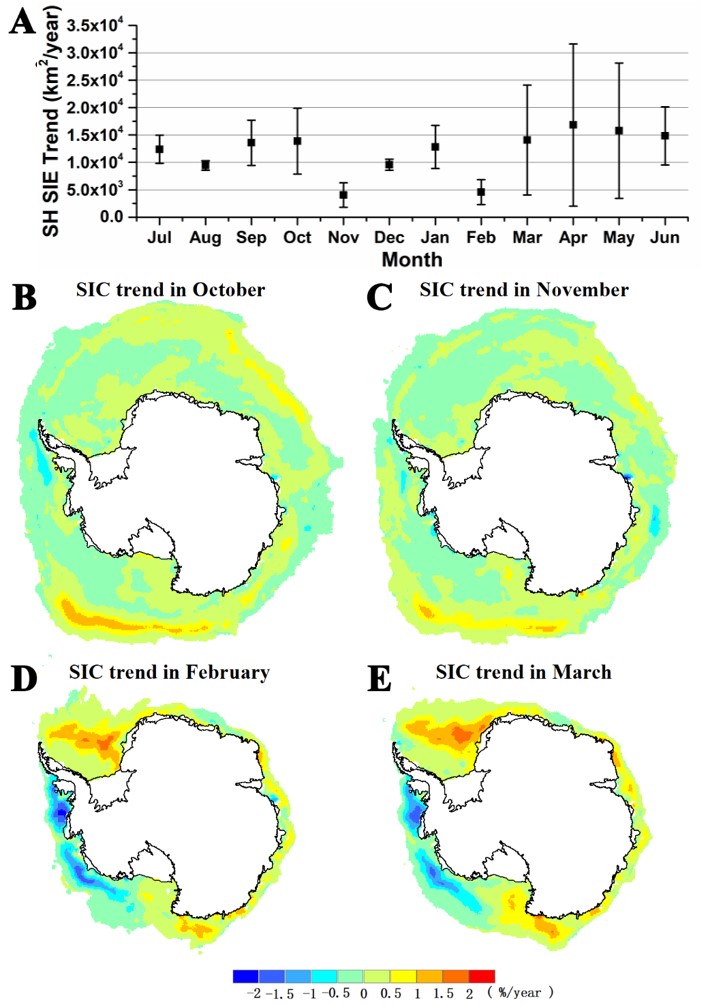


**Figure S2.** (A) Trend of sea ice extent (SIE, defined as sum of ice-covered area where sea ice concentration > 15%) in each month and spatial pattern of trend of sea ice concentration (SIC) in (B) October, (C) November, (D) February and (E) March, 1978-2020

**
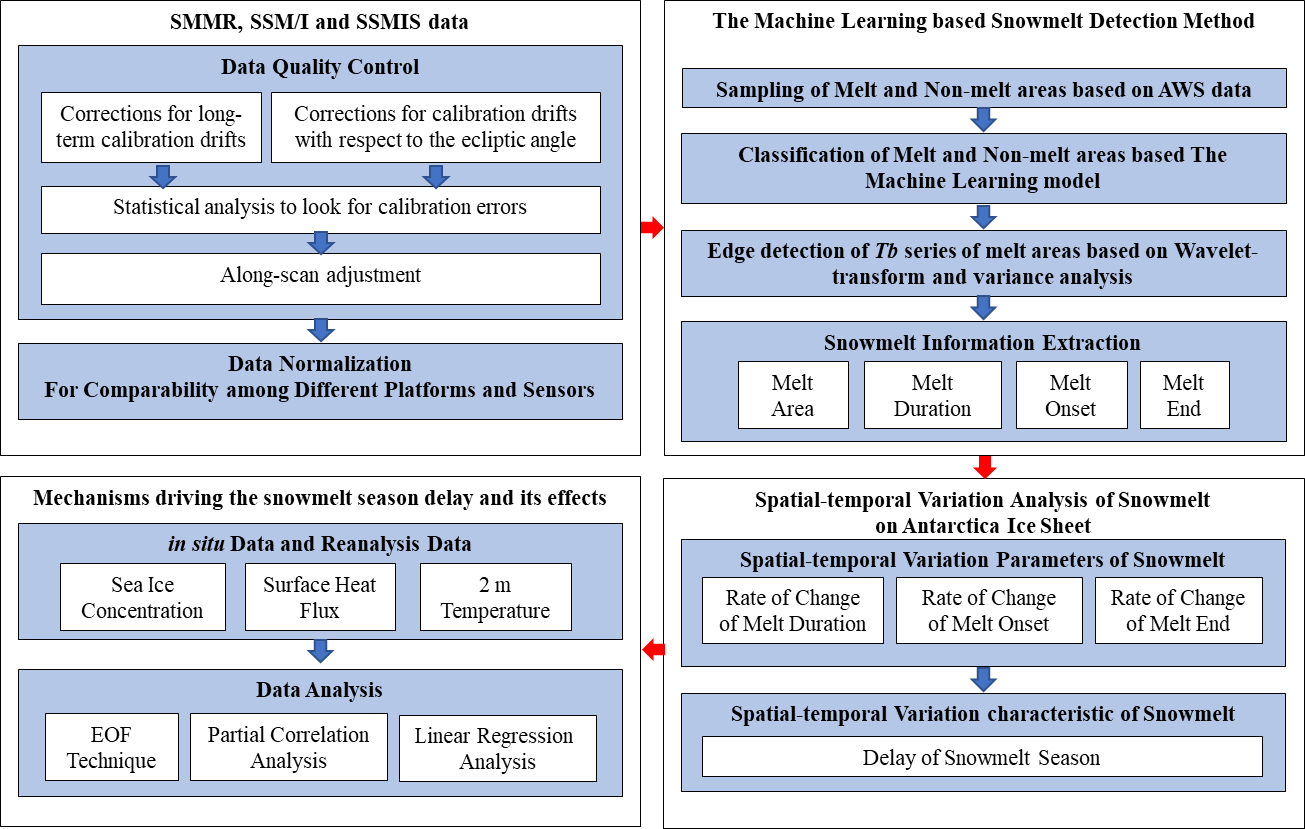
**

**Figure S3.** Research framework in the study of delay of Antarctica snowmelt season. (1) Tb Data acquisition. One of the longest and reliable microwave radiometer data records are used to snowmelt detection on Antarctica ice sheet; (2) Ice sheet snowmelt detection. The Machine Learning based Snowmelt Detection Method and an appropriate sampling scheme are applied to detect the ice sheet snowmelt; (3) Spatial-temporal variation analysis of snowmelt. Three new parameters are proposed to reveal the variation characteristics in the timing of the Antarctic ice sheet melt. (4) Mechanism explanations of delay of Antarctica snowmelt season and its effects. Three main factors, atmosphere, sea ice and sea water, are exploited to investigate the reason of the delayed phenomenon.


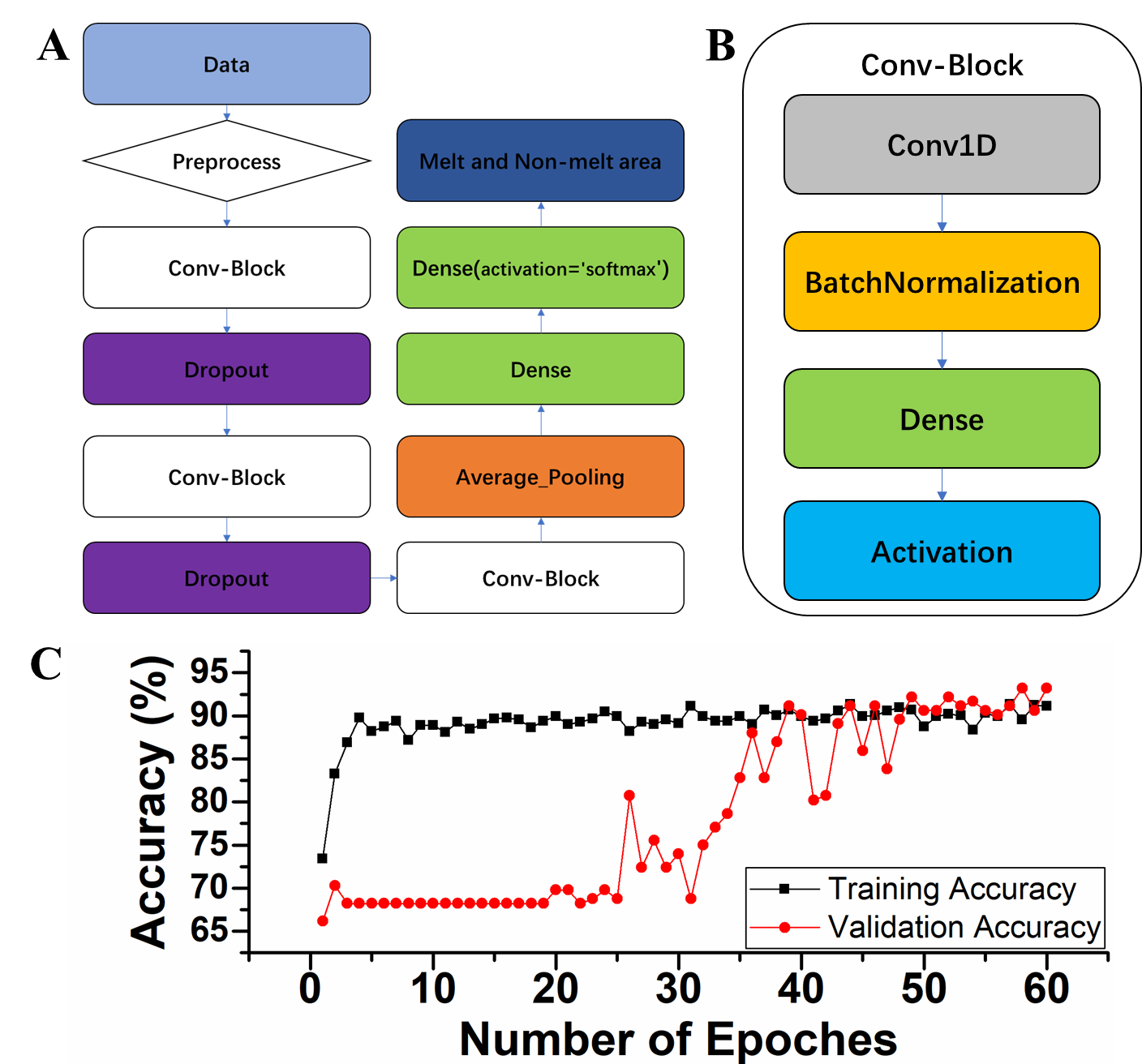


**Figure S4.** (A) The architecture of the artificial neural network; (B) Details of the Conv-Block; (C) The classification accuracy of melt and non-melt area based on the machine learning method. The training data accuracy is plotted in black and the validation data in red.

**Figure S5.** Brightness temperature variation and correspond to air temperature in AWS.

**Figure S6.** Train accuracy and test accuracy with the different convolutional layers setting and the percentage of train and test samples.


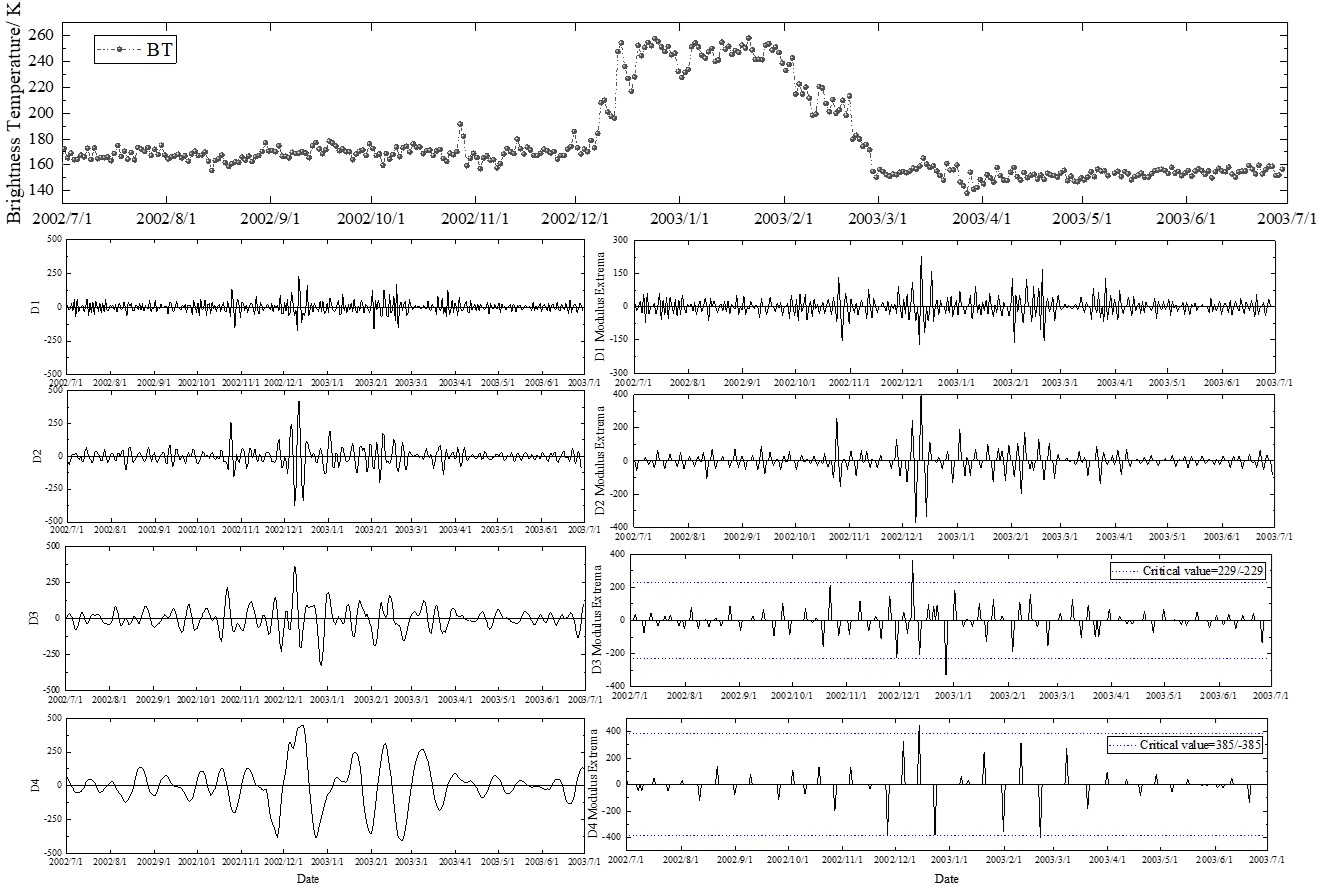


**Figure S7.** Multi-scale decomposition of Tb time series by a fast discrete wavelet transform. Original daily brightness temperature and wavelet transform and modulus extrema of the original brightness temperature at multi-scales.


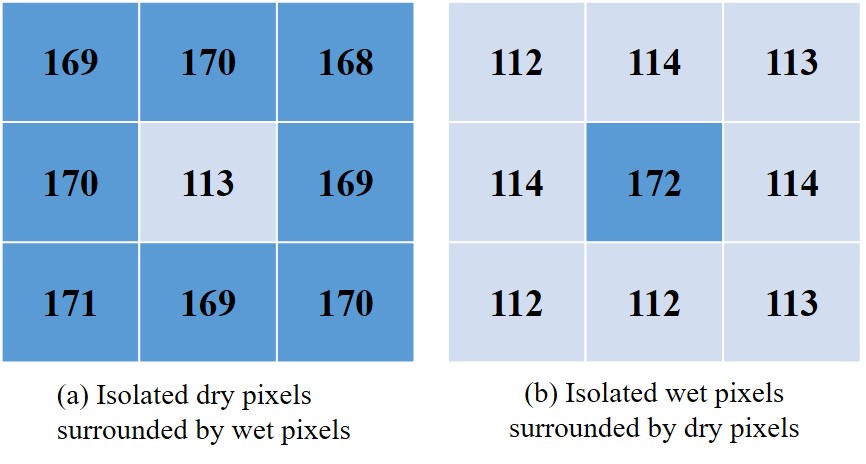


**Figure S8.** The noise pixels. (a) isolated dry pixels surrounded by wet pixels; (b) isolated wet pixels surrounded by dry pixels


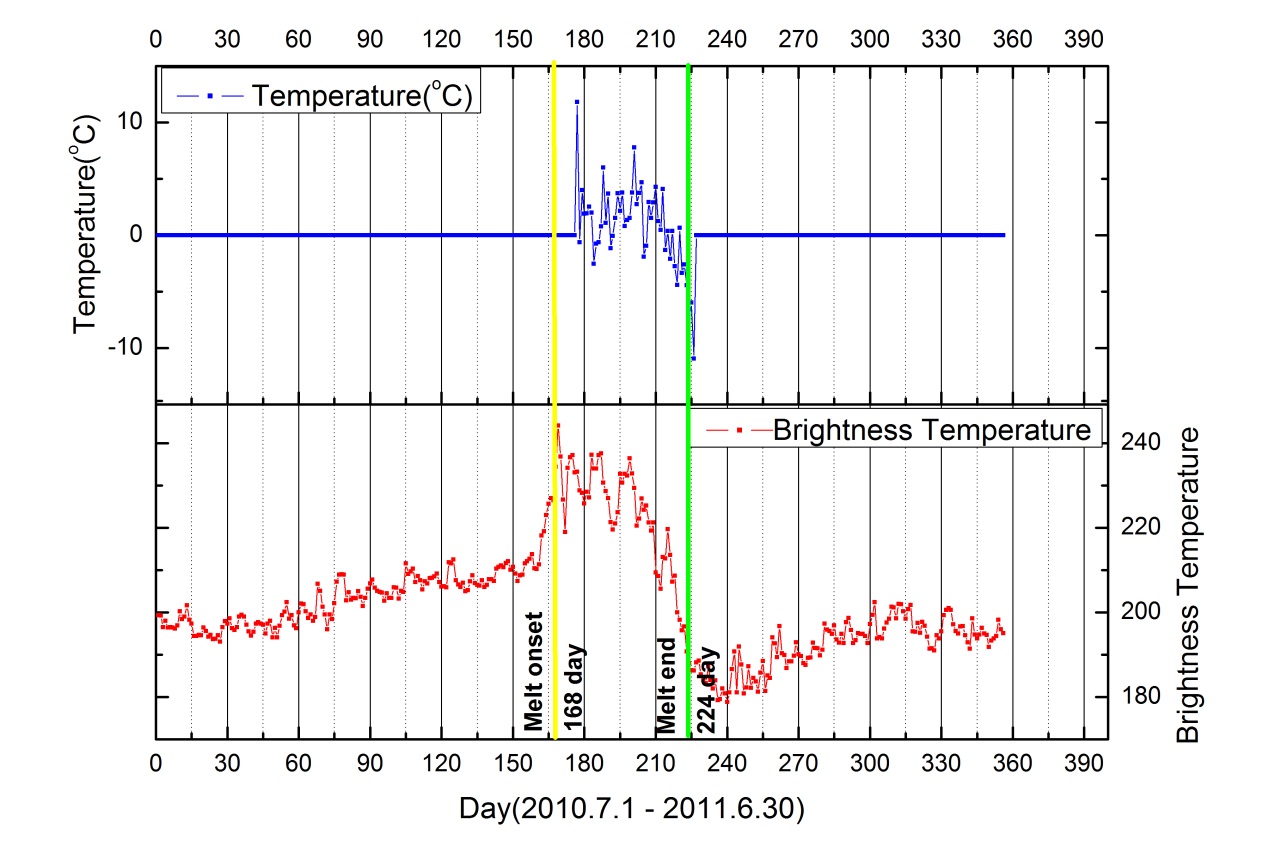


**Figure S9.** In situ data from 27th Chinese national Antarctic expeditions for the validation of detection of ice sheet snowmelt date on Zhongshan station.


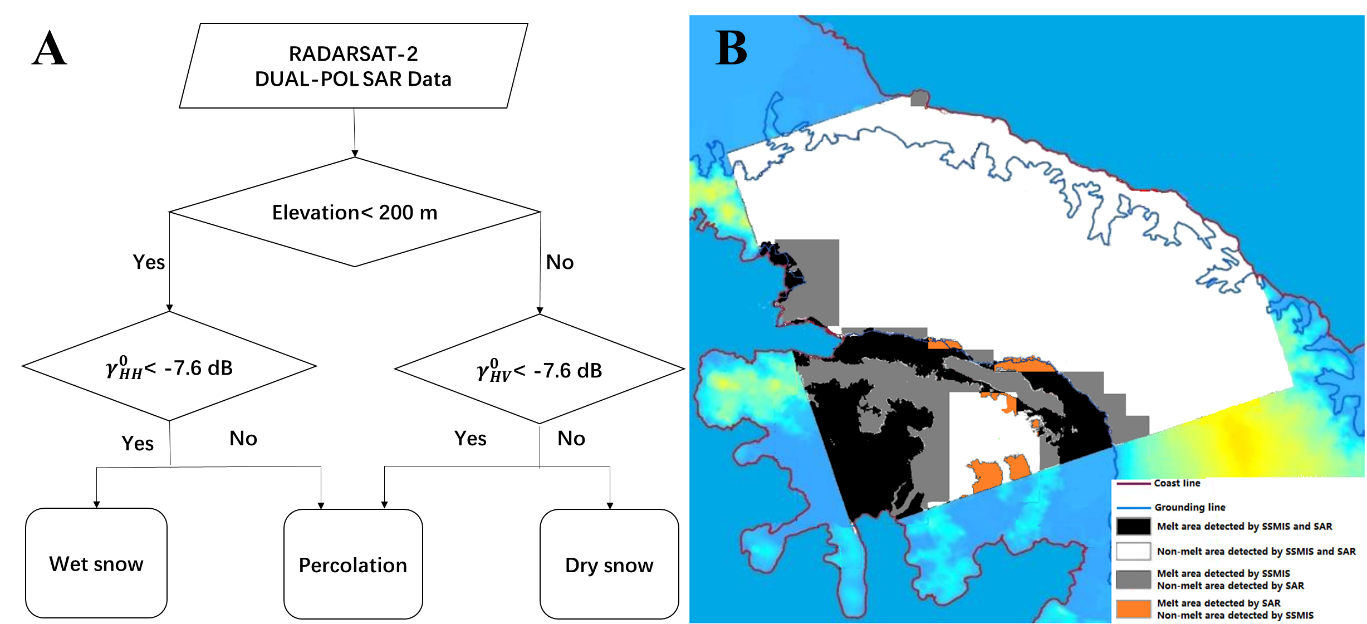


**Figure S10.** (A) The decision classification tree of the algorithm used to determine ice sheet melt in the Radarsat scene shown in panel B. $\gamma_{HH}^{o}$and$\gamma_{HV}^{o}$are the horizontal and cross polarization channel backscattering coefficients respectively. (B) Comparison of melt detection result from SMMIS radiometer data and the RADARSAT-2 Dual-Pol SAR image from 25th Jan. 2010. The black and white areas show the region where the SSMIS data using our method and SAR data are agreement. The melt area detected using our method is 66,875 km2, the one detected by SAR data is about 42,144 km2 and the overlapping melt area detected by both is 40,254 km2. Possible reasons for the difference in areas are discussed in Validation section of the SI Discussion.


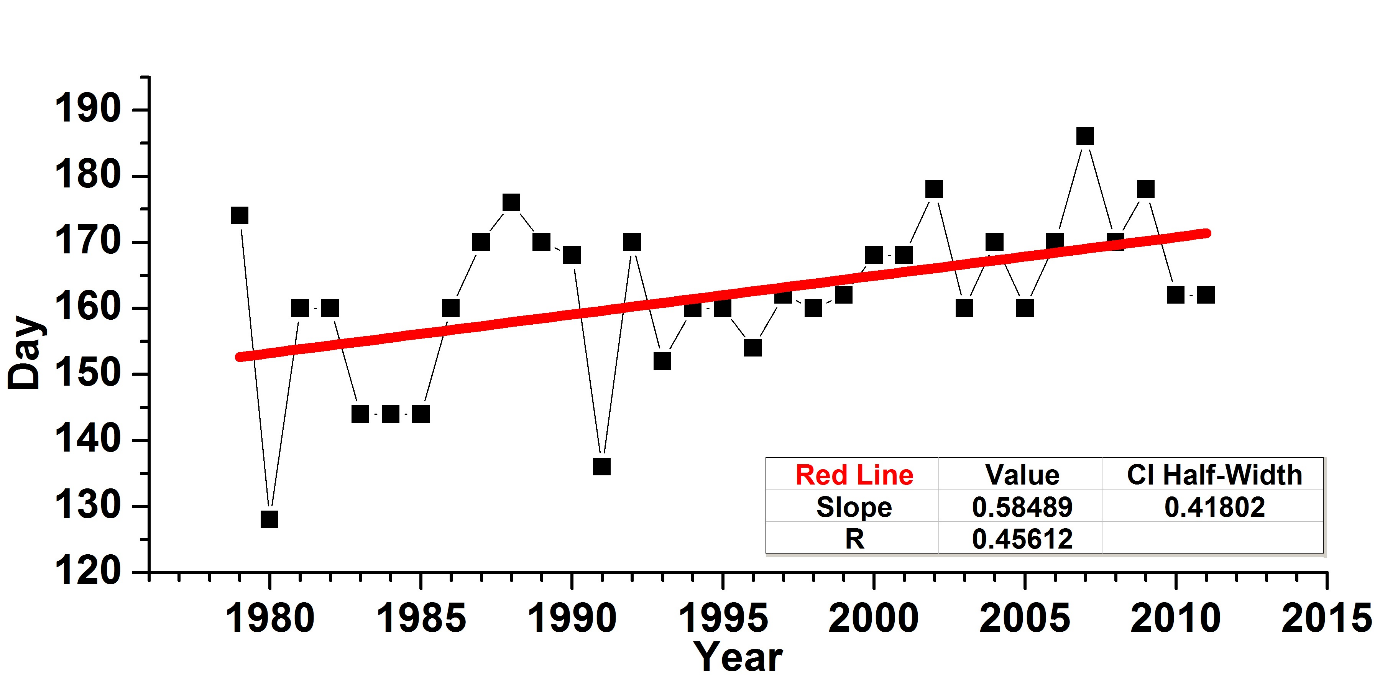


**Figure S11.** Change rate of the Antarctic melt onset date.

**Table S1.** Automatic weather stations for the validation of delay of ice sheet snowmelt season

| No. | Station name | Latitude/Longitude | Dates |
| --- | --- | --- | --- |
| 1 | Arturo Prat | -62.5S/-59.7 | 1979-2020 |
| 2 | Belgrano II | -77.9S/-34.6 | 1979-2020 |
| 3 | Bellingshausen | -62.2S/-58.9 | 1979-2020 |
| 4 | Casey | -66.3/110.5 | 1979-2020 |
| 5 | D-10 | -66.71/139.84 | 01/1980-12/1980  01/1983-12/1985  01/1980-12/1980  01/1987-12/1989  01/1991-12/1994  01/1998-12/2002  01/2009- |
| 6 | Davis | -68.6/78 | 1979-2020 |
| 7 | Dumont Durville | -66.7/140 | 1979-2020 |
| 8 | Esperanza | -63.4/-57 | 1979-2020 |
| 9 | Faraday\Vernadsky | -65.4/-64.4 | 1979-2020 |
| 10 | Gill | -79.93/-178.59 | 01/1986-12/1988  01/1990- |
| 11 | Great Wall | -62.2/-59 | 1979-2020 |
| 12 | Grytviken | -54.3S/-36.5 | 1979-2020 |
| 13 | Halley | -75.5/-26.4 | 1979-2020 |
| 14 | Larsen Ice Shelf | -66.90/-60.60 | 02/1983- |
| 15 | Manuela | -74.95/163.69 | 01/1985-12/1985  01/1987-12/2003  01/2005-12/2005  01/2009- |
| 16 | Marambio | -64.2/-56.7 | 1979-2020 |
| 17 | Marble Point | -77.44/163.76 | 01/1985- |
| 18 | Marsh | -62.2/-58.9 | 1979-2020 |
| 19 | Mawson | -67.6/62.9 | 1979-2020 |
| 20 | McMurdo | -77.9/166.7 | 1979-2020 |
| 21 | Mirny | -66.5/93 | 1979-2020 |
| 22 | Neumayer | -70.7/-8.4 | 1979-2020 |
| 23 | Novolazarevskaya | -70.8/11.8 | 1979-2020 |
| 24 | O Higgins | -63.3/-57.9 | 1979-2020 |
| 25 | Orcadas | -60.7/-44.7 | 1979-2020 |
| 26 | Palmer | -64.3/-64 | 1979-2020 |
| 27 | Pegasus North | -77.96/166.51 | 1979-2020 |
| 28 | Rothera | -67.5/-68.1 | 1979-2020 |
| 29 | San Martin | -68.1/-67.1 | 1979-2020 |
| 30 | Scott Base | -77.9/166.7 | 1979-2020 |
| 31 | Syowa | -69/39.6 | 1979-2020 |
| 32 | Zhongshan | -69.4/76.4 | 1979-2020 |

**Table S2.** Automatic weather stations for the evaluation of the snowmelt detection methods.

| Station name | Latitude/Longitude | Elevation | Dates | Days |
| --- | --- | --- | --- | --- |
| Larsen Ice Shelf | 66.90S/60.60W | 50 m | 02/1983- | 2390 |
| Butler Island | 72.20S/60.34W | 91 m | 03/1986- | 2449 |
| Mount Siple | 73.198S/127.052W | 8m | 01/1992- | 1761 |
| Amery G3 | 70.892S/ 69.873E | 84 m | 01/1999- | 1931 |
| Lanyon | 66.278S/110.797E | 390m | 01/1991-12/2008 | 2015 |
| Cape Bird | 77.224S/166.440E | 38m | 01/1999-12/2002  01/2009- | 1845 |
| Limbert | 75.420S/59.850 | 59m | 01/1995-12/1997  01/2000-12/2002  01/2009- | 2111 |

**Table S3.** Quantitative snowmelt detection results (in %)

| Automatic weather station | Snowmelt detection method | Data | *^&^P_TP_* | *^&^P_TN_* | *^&^*CDR |
| --- | --- | --- | --- | --- | --- |
| Mount Siple | The improved method | SMMR, SSM/I, SSMIS | 61 | 22 | 83 |
|  | The improved method | AMSR-E | 53 | 24 | 77 |
|  | *^#^*XPGR | SMMR, SSM/I, SSMIS | 55 | 24 | 79 |
| Butler | The improved method | SMMR, SSM/I, SSMIS | 8 | 73 | 81 |
|  | The improved method | AMSR-E | 5 | 75 | 80 |
|  | XPGR | SMMR, SSM/I, SSMIS | 4 | 72 | 76 |
| Larsen Ice Shelf | The improved method | SMMR, SSM/I, SSMIS | 32 | 53 | 85 |
|  | The improved method | AMSR-E | 32 | 44 | 76 |
|  | XPGR | SMMR, SSM/I, SSMIS | 28 | 50 | 78 |
| Amery G3 | The improved method | SMMR, SSM/I, SSMIS | 5 | 83 | 88 |
|  | The improved method | AMSR-E | 5 | 74 | 79 |
|  | XPGR | SMMR, SSM/I, SSMIS | 6 | 72 | 78 |
| Lanyon | The improved method | SMMR, SSM/I, SSMIS | 45 | 35 | 80 |
|  | The improved method | AMSR-E | 42 | 30 | 72 |
|  | XPGR | SMMR, SSM/I, SSMIS | 39 | 34 | 73 |
| Cape Bird | The improved method | SMMR, SSM/I, SSMIS | 9 | 70 | 79 |
|  | The improved method | AMSR-E | 9 | 69 | 78 |
|  | XPGR | SMMR, SSM/I, SSMIS | 9 | 70 | 79 |
| Limbert | The improved method | SMMR, SSM/I, SSMIS | 30 | 52 | 82 |
|  | The improved method | AMSR-E | 24 | 51 | 75 |
|  | XPGR | SMMR, SSM/I, SSMIS | 27 | 48 | 75 |

*^&^*Defined in the Supplementary Note S1, Method Validation section. *^#^*See reference (46) in the main text.

**Supplementary Note S1.**

**Characteristics of snowmelt detection based on Microwave Radiometer Method and extraction of signal**

Grain size, density, crystal structure and surface conditions (hoar frost, layering or crusts produced by wind or radiation) of snow and ice all contribute to the emissivity of snowpack, However the temporal scale at which the appearance of liquid water affects the brightness temperature (*Tb*) is much shorter than that of these other factors. In other words, the presence of liquid water within the snowpack leaves a clear (significant increase) and distinct (the increase occurs from one day to another or even at shorter timescales) signature on passive microwave observations. Changes in the liquid-water content produce the most prominent variations in the emissivity (*e*) and hence in the *Tb*. Liquid water may come from rainfall or snowmelt. In the absence of rainfall which is extremely rare in Antarctica, liquid water comes from snowmelt generated by air temperature and solar radiation. Therefore, a small amount (a few percent by volume) of liquid water induced by the melting process can radically increase the microwave emissivity of a snowpack. Therefore, the transition of dry snow to wet snow (liquid water, ice and air) yields a distinct signature: a sharp and abrupt increase in *Tb*, which is detectable by microwave sensors at frequencies in excess of 10 GHz.

We chose the 19.35 GHz frequency, instead of others also available from SSM/I (e.g., 37 GHz), for two reasons: first, data measured at 37 GHz are more influenced by fluctuations of surface parameters (e.g., grain size) than 19.35 GHz data; second we observed that, in agreement with other studies, brightness temperature at 19.35 GHz, horizontal polarization shows the largest difference between melt onset and dry snow conditions, hence providing more sensitivity to melting events (SI reference 1 and the references therein; and references (19, 43) in the main text). In this way, we can separate melt or refreezing events from the precipitation signal, the change in grain size, etc.

Above all, the use of microwave radiometers to detect liquid water in the upper snowpack is robust.

The relationship between the *Tb* and near-surface physical temperature *T_s_* and *e* of snow and ice can be represented by the first-order Rayleigh–Jeans approximation (SI reference 2):

*Tb = eT_s_*  （1）

When the *Tb* time series is plotted as a one-dimensional curve, the structures with an edge (peak) shape are present at melting and refreezing times. Similarly to Joshi et al. (SI reference 3), we exploit the fact that the occurrence of strong and significant edges in the *Tb* time series curve signifies snow melting and refreezing events, and hence we can determine when a pixel experienced melt. Moreover, through variance analysis in our method, an optimal edge strength threshold is statistically determined to differentiate real snowmelt edges from weak edges caused by noisy perturbations and other non-melt processes.

The percolation zone is the area that experiences some degree of melt, but not to the point of saturation of the previous winter accumulation. Since our method is sensitive to degree of melt it detects areas that experience melting on the Antarctica ice sheet, whether or not they occur on the percolation zone.

**Snowmelt Detection Method based on Machine Learning and Wavelet-transform**

This study proposed a snowmelt detection method based on machine learning jointed wavelet-transform approach. This method involves two key processes. One is the identification of dry and wet snow pixels, and another is to determine the date of thaw and freeze signal occurs in a year. Therefore, the uncertainty of snowmelt detection method proposed by this study generally originate from classification for dry and wet snow pixel and wavelet-transform based on edges detection.

**1) Classification for dry and wet snow pixel based on machine learning**

Although the machine learning provides a valid means for the classification of snowmelt and non-snowmelt, the results contain some uncertainties result from the machine learning model, training samples and parameter setting. A neural network with three convolution blocks combined with fully connected layers is applied. Max pooling layer is used in our CNN model, which helps reduce the spatial size of the convolved features and also helps reduce over-fitting by providing an abstracted representation of them. The activation used in each convolutional block is the RelU activation function. The earning rate is 0.01 and the ADAM optimizer is used. The SoftMax function is used in the last layer of the convolutional neural network to classify the data. The air temperature observed by ten AWS stations in different location over Antarctic were regarded as the proxy of surface melt and non-melt. A total of 956 samples were selected, which consist of the labels determined by the corresponding temperature data based on an air temperature threshold and the 18/19 GHz horizontal polarization channel brightness temperature data of the SSMR and SSM/I and SSMIS.

The results of machine learning methods are strictly dependent on the quality of the training samples. In general, the snow melt onset date generally corresponds to the occurrence of the air temperature above or near the melting point (0℃). However, it is worthy to note that the occurrences of positive air temperature from weather stations do not always correspond to satellite-derived snowmelt due to the inconsistency in spatial scales (i.e., representativeness errors). There is an example in the Larsen Ice Shelf station in Figure S5. We can see that the atmospheric temperature is greater than the melting point on November 9, 21, and 24, 2002, but there is no melting event actually occurred at this time. In addition, Antarctic surface melting is driven by a variety of processes such as solar radiation, latent heat, and sensible heat energy exchange, which is a complex issue. Snowmelt may occur when air temperature is below the freezing point but the snowpack is considerably warmed by solar radiation, such as the Gill station from November to March in following year. Although it is not rigorous to distinguish melt and non-melt only by atmospheric temperature, it can be used as an option in the absence of field verification data. The melt point temperature used to distinguish melt from non-melt pixels that are regarded as the training sample will bring some uncertainty to the results. Additionally, we considerate a more refined sampling scheme in order to keep the melt and non-melt sample balanced. The sampling principle of dry snow and wet snow was proposed, which is that the extraction of fewer dry snow samples in stable regions and more samples in unstable regions. Since dry snow is distributed over a much wider area of Antarctica than wet snow, we use disproportionate sampling to improve sample representativeness and enhance reliability leading to a general principle: a small number of samples collected in areas where climate is relatively stable, such as East Antarctica, inland, or high-latitude and -altitude areas, and a larger number of samples in areas of complex climatic variability, such as West Antarctica, coastal areas, low-latitude and -altitude areas and ice shelves where climate varies considerably.

Moreover, the CNN architecture, convolutional layers setting and training epochs can affect the performance for snowmelt detection, which will inevitably bring some errors to the melt onset, end and duration. We analyzed the model train and test accuracy with the different convolutional layers setting and the percentage of train and test samples in the training process. As seen in Figure S6, the train and test accuracy were all above 90% indicating the better performance and stability of CNN model classification of snowmelt. The effect of percentage of train and test samples in the training process and the convolutional layers on classification results were slightly. In order to improve processing efficiency for Antarctic ice sheet, the 80% of samples finally were used for training, and 20% for test. We set up three convolutional layers in our CNN model.

2) **Wavelet-transform based on edges detection**

In this study, we adopt a wavelet transform based approach to edge detection. Thus, the decomposed scale of bright temperature signals and critical value to determine the location of modulus maxima both may bring the uncertainty of melt onset, end and duration.

First, the passive microwave BT signals are decomposed into multi-scale components that are well localized in both time and frequency through the wavelet transform. However, the decomposed scale will directly affect the location detection of melt onset and end, thereby the duration. Figure S7 shows the 19 H BT variation form July 1, 2002 to June 30, 2003 in Larsen ice shelf and the corresponding wavelet transform results across multi-scales. Less decomposed scales can generate more modulus extreme quantities, which may interfere the snowmelt detection and if the critical value is lower resulting in the overestimate of melt duration. We selected the scale 2^4^ that it is sufficient to decompose the BT time series (SI reference 5) and the uncertainty of snowmelt detection can be reduced using the appropriate decomposed scale. 2^3^ scale obtained the melt onset is December 8,2002 and melt end is December 22, 2002 and melt duration is 20 days. However, the detection of melt onset, end and duration are December 15, 2002, February 2, 2003, and 69 days, respectively by 2^4^ scale. We referred to the wavelet transform scale by Liu at el. (2005) (SI references 5), which extensive experiments show that it is sufficient to decompose the BT time series up to the scale 2^4^, because the wavelet components at the scale larger than 2^4^ contain only the information regarding the general seasonal trend of brightness temperature variations. The melt and refreeze signals are contained in the decomposed wavelet components at the scale less than 2^4^. Therefore, 2^3^ scale was selected in our study.

Second, the critical value also directly affects melt onset, end and duration. The optimal threshold of wavelet analysis may not fully adapt to snow cover in all regions. We used variation analysis method to identify a critical value as a dividing point to classify the local extrema of wavelet transform modulus into two groups: a lower maxima group and an upper maxima group the wet snow pixel. The selected critical value is finally used to determine the location of wavelet transform modulus extrema in wet snow pixel. The computation results from the wavelet-transform based edge detection may be sensitive to the critical value, and a slight overestimate or underestimate of the critical values may cause incorrect outputs. Finally, for the daily BT, time series may be contaminated and distorted in some conditions, such as strong sensor noise, clouds, geolocation errors and the existence of ponding water or strong relief within a data pixel, which result in the uncertainty of the snowmelt detection results. The noise pixels often come into sight as isolated wet pixels surrounded by dry pixels, or isolated dry pixels surrounded by wet pixels (As seen in Figure S8). We deal with these situation using a spatial neighbourhood operator to detect and correct possible errors brought about by noise pixels and automatically flag potential errors. Each pixel was examined with a 3×3 window for reducing the difference of melt onset, end and duration between this pixel and surrounding pixels. Additionally, there is another situation is only for the wet pixels whose melt onset date, melt end date or melt duration are substantially different from those of their neighbouring pixels. We reduce errors by adjusting the critical values.

**Method Validation**

We validate our melt detection results with coincident near-surface air temperature data at several automated weather stations. Later we also evaluate our melt detection result by comparison with the melt results obtained by SAR. The evaluation (Table S3) demonstrates that our method does measure the right thing well.

The In situ data on Zhongshan station measured by the 27^th^ Chinese national Antarctic expeditions is used for the validation of detection of ice sheet snowmelt date. Fig. S9 show the daily mean air temperature data together with the corresponding daily detection results of the new snowmelt detection method. The green line indicate that the melt end date detected by the method agrees with the date when the temperature begins to drop to below zero. From this result of the method, it demonstrated that the snowmelt detection of our method is effective. In addition, we use in situ data from AWSs with more than 10 years of observations to validate the daily snowmelt detection method. Since AWS do not cover the same area observed by large-scale remote sensing they do not quite measure the same thing as our method. Therefore, to minimize the influence of the environment, we choose AWSs located areas where the spatial gradients are relatively spatially uniform and hence their temperature data should reflect average temperatures over large areas. We used the Radarsat Antarctic Mapping Project Digital Elevation Model, 1-km data to calculate the standard deviation, minimum and maximum heights in a 625 km^2^ area around the AWS to determine spatial complexity of the terrain, selecting AWS stations shown in Table S2 (the AWS can be obtained from <http://amrc.ssec.wisc.edu/aws/>).

The daily mean air temperature data together with the corresponding daily detection results of the new snowmelt detection method were used to quantitatively validate the snowmelt detection results of the method. For processing the AWS data, we chose the daily mean air temperature data from the summer months (November–March) in Antarctica. The total number of days *N* (i.e., the total number of daily mean air temperature data) chosen in each AWS can be seen in Table S2. The daily mean air temperature data are made up of the mean of three daily maximum air temperatures; a daily mean maximum air temperature above 0°C is regarded as a snowmelt occurrence. A melt day is the day with daily mean maximum air temperature is above 0°C and a non-melt day is the day whose daily mean maximum air temperature is below 0°C. We compare the results from the AWS and our method using:

1) Fraction of true positives (TP), that is the melt days that were correctly detected, *P_TP_=TP/N*

2) Fraction of true negatives (TN), that is non-melt days that were correctly detected, *P_TN_=TN/N*

3) Correct Detection Rate (CDR), the ratio of correct detection results to the total number of detection results (or the total number of temperature data), *CDR=(TP+TN)/N*

The statistical analysis is shown in Table S3. The *TP* values of the method are very small near AWS Butler, Amery G3 and Cape Bird, 8% 5% and 9% respectively. This is because the number of melt days recorded by these AWS are very few, accounting for only 10 %, 6% and 8% of their respective records, and so TN needs to also be considered, as it is in the CDR statistic. Most CDR exceed 80%, indicating that new method is effective.

Table S3 also shows for how the method and data set we use in the manuscript compares with our method using AMSR-E data and how it compares with the XPGR method (references (46) in the main text). In both cases the method and data we use out-performs the others.

Moreover, we compared our results with the estimate using a RADARSAT-2 Dual-Pol SAR image. The SAR-based melt detection process is shown in Fig. S10 A. Fig. S5B shows that the melt detections are consistent. However, there are differences in two snowmelt detection results, because: (1) difference in resolution of the two datasets; (2) the specific acquisition time is different - the acquisition time of radiometer data is 5:00 pm and the one of SAR data is 7:00 am. So the physical state of the snow surface may be different at the two different time; (3) the incidence angle is different: the incidence angle of the radiometer is 53.1° and the SAR data is from 20° to 49°, this affects the acquisition data as topography is not flat in this area; (4) the radiometer uses 18 GHz or 19 GHz and SAR 5.4 GHz, so the snow penetration depth is different.

Snow melt is difficult to detect using optical imagery. There are two reasons: (1) cloud-free conditions are rare in polar regions; (2) it is hard to distinguish dry snow and wet snow with low water content.

**Supporting References**

1. Tedesco M. Assessment and development of snowmelt retrieval algorithms over Antarctica from K-band spaceborne brightness temperature (1979–2008). *Remote Sens of Environ* 2009; **11**: 3979–97.
2. Foster JL, Hall DK and Change TC *et al*. An overview of passive microwave snow research and results. *Reviews of Geophys and Space Res* 1984; **22**: 195–208.
3. Joshi M, Merry CJ and Jezek KC *et al*. An edge detection technique to estimate melt duration, season and melt extent on the Greenland ice sheet using passive microwave data. *Geophys Res Lett* 2001; **28**: 3497–500.
4. Liu HX, Wang L and Jezek KC. Wavelet-transform based edge detection approach to derivation of snowmelt onset, end and duration from satellite passive microwave measurements. *Int J Remote Sens* 2005; **26**: 4639-60.
